# Right Atrial Vegetation on a Chiari Network Presenting With Multifocal Pulmonary Lesions

**DOI:** 10.7759/cureus.108591

**Published:** 2026-05-10

**Authors:** Dharmikkumar Jadvani, Rutvikkumar Jadvani, Aakash Rana, Jack Xu

**Affiliations:** 1 Internal Medicine, University of Arkansas for Medical Sciences, Little Rock, USA; 2 Internal Medicine, Government Medical College, Surat, IND; 3 Medicine, Central Arkansas Veterans Healthcare System, Little Rock, USA; 4 Cardiology, Novant Health, Winston-Salem, USA

**Keywords:** cardiac vegetation, chiari network, infective endocarditis, lung embolus, nonvalvular endocarditis, rare cardiac infection, right atrial cardiac mass, vegetation

## Abstract

The Chiari network is a rare embryological fenestrated web-like structure located in the right atrium, present in approximately 2-3% of the general population. Infective endocarditis of the Chiari network is exceedingly rare and poses significant diagnostic and management challenges. We present a case of a 67-year-old male with hypertension, chronic obstructive pulmonary disease (COPD), and chronic hepatitis C who presented with malaise and dyspnea. Workup revealed multifocal bilateral pulmonary opacities on computed tomography (CT) of the chest, and transthoracic echocardiography (TTE) identified a right atrial echodensity of uncertain significance. The infectious workup was unrevealing. Transesophageal echocardiography (TEE) subsequently demonstrated a new oscillating echodensity on the Chiari network, leading to a diagnosis of Chiari network endocarditis. The patient was treated empirically with a six-week course of intravenous antibiotics. Our case highlights the importance of advanced cardiac imaging in diagnosing Chiari network endocarditis and embolic pulmonary findings.

## Introduction

The Chiari network is an embryonic remnant of the right valve of the sinus venosus, appearing as a thin, fenestrated, web-like structure within the right atrium [1]. It is present in approximately 2-3% of the general population, originating from the eustachian and thebesian valvular regions, attached to the upper wall of the right atrium or atrial septum [2]. Although generally considered a benign anatomical variant, it can serve as a nidus for thrombus formation and cause complications such as thromboembolic disease, cardiac arrhythmias, entrapment of intracardiac devices, and, rarely, infective endocarditis [2]. Among the very few reported cases of Chiari network endocarditis, isolated Chiari network involvement is extremely rare [3].

Here, we present a case of a 67-year-old male with a history of hypertension, chronic obstructive lung disease (COPD), and prior methicillin-sensitive *Staphylococcus aureus *(MSSA) spinal cord stimulator infection who developed endocarditis of the Chiari network with septic pulmonary emboli. Our case highlights the diagnostic challenges posed by transthoracic echocardiography's (TTE) limited ability to characterize the lesion and the indispensable role of transesophageal echocardiography (TEE) in achieving definitive visualization. Our case is unique as it is exclusively limited to the involvement of the Chiari network. The aim of this report is to raise awareness of this uncommon entity and utilize the diagnostic value of multimodal cardiac imaging.

## Case presentation

A 67-year-old male with a past medical history significant for hypertension, COPD, and chronic hepatitis C presented on June 27 with a one-and-a-half-week history of progressive weakness and shortness of breath. His relevant past medical history included a spinal cord stimulator infection caused by MSSA in 2020, which was treated at that time, and partial treatment of hepatitis C in 2020, with completion of therapy in March 2023.

On arrival, his temperature was 98.5°F. Initial laboratory evaluation revealed a white blood cell count of 12.1 × 10^9^/L (reference range: 5-10 × 10^9^/L), consistent with mild leukocytosis. The patient reported chronic back and hip pain that had been present since his 2020 MSSA spinal infection and had remained stable. He denied intravenous (IV) drug use, recent skin infections, and recent dental procedures. Social history was notable for smoking a few cigarettes per day and consuming one to two cans of beer per day.

Physical examination revealed raised, erythematous, pruritic plaques on the lower extremities, which had begun around the time of his initial malaise. These lesions had partially improved since onset but were beginning to recur at the time of presentation. Decreased breath sounds were noted on auscultation. Spinal tenderness was absent on palpation.

**Figure 1:**
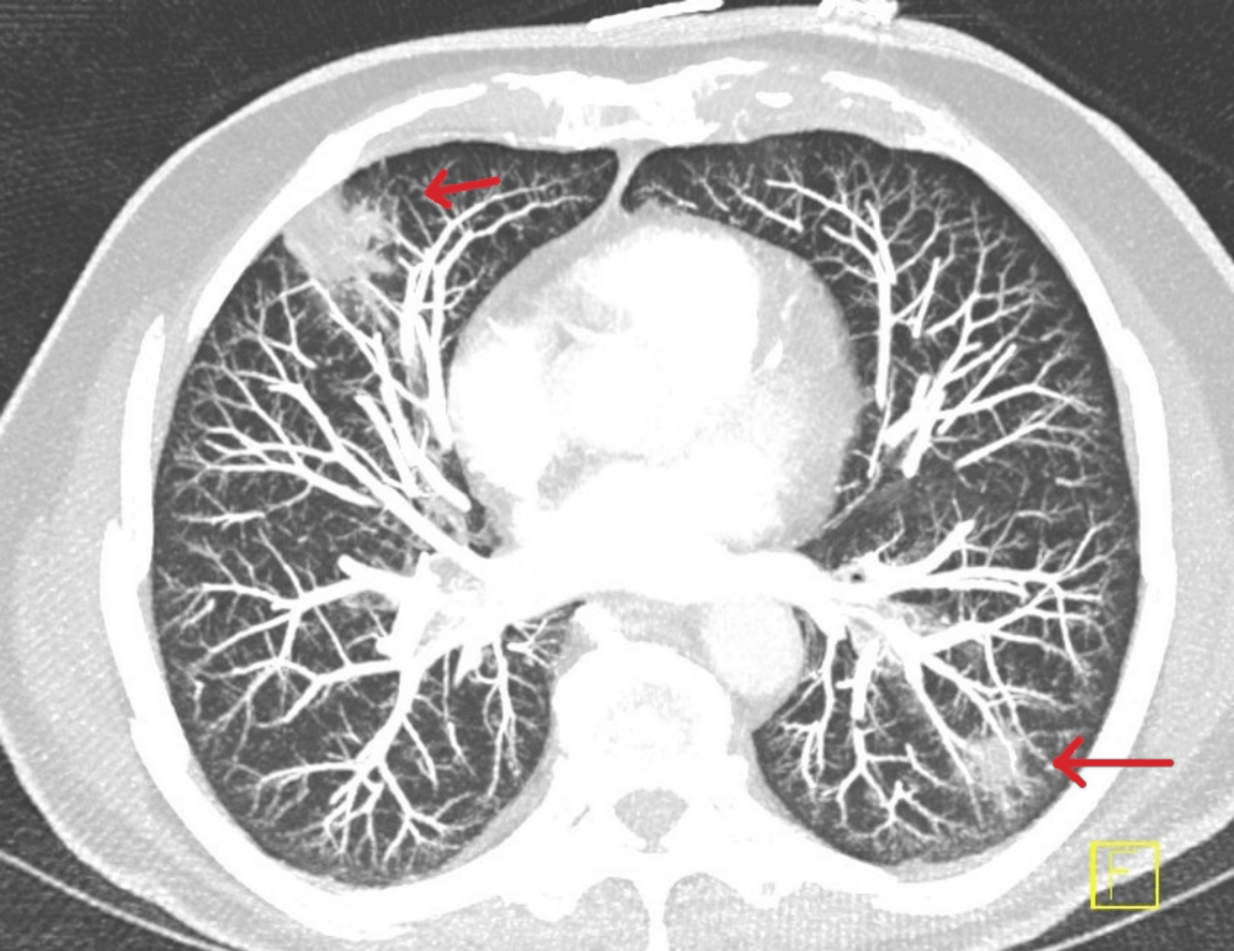
Contrast-enhanced computed tomography (CT) of the chest demonstrating multifocal bilateral peripheral nodular and mass-like opacities Contrast-enhanced CT of the chest demonstrating multifocal bilateral peripheral nodular opacities (red arrows), consistent with septic pulmonary emboli in the setting of right-sided infective endocarditis.

A 12-lead electrocardiogram (ECG) demonstrated a normal sinus rhythm without evidence of acute ischemia, myocardial infarction, or conduction abnormality. Blood cultures drawn at admission showed no growth at five days. Computed tomography (CT) angiogram of the chest demonstrated multifocal bilateral nodular/mass-like pulmonary opacities (Figure 1).

The differential diagnosis based on CT findings included multifocal pneumonia from an embolic source, organizing pneumonia, and pulmonary malignancy or metastatic disease. CT of the abdomen and pelvis was unremarkable.

TTE was performed to evaluate for a cardioembolic source. TTE demonstrated normal biventricular systolic function with a left ventricular ejection fraction (LVEF) of 55-60% (Video 1).

**Video 1:** Transthoracic echocardiography (TTE) showing a linear, mobile echogenic structure in the right atrium, suggestive of a Chiari network with possible superimposed vegetation

**Figure 2:**
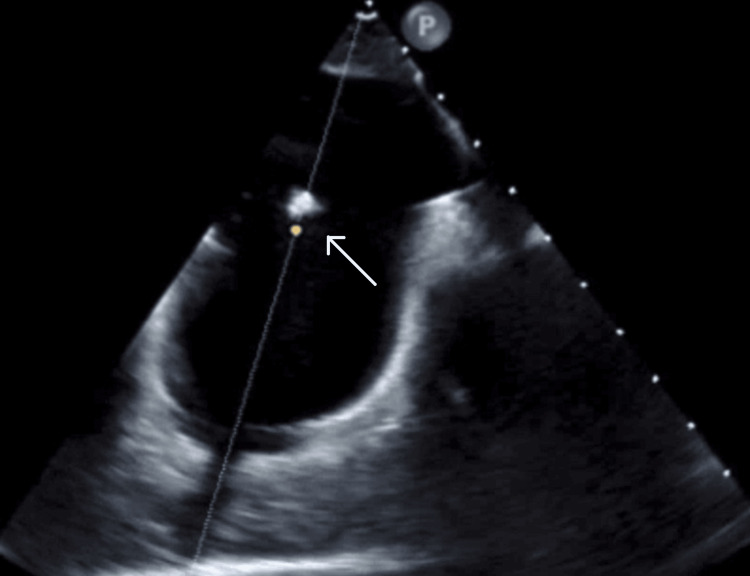
X-plane transthoracic echocardiography (TEE) showing echodensity attached to Chiari network Transthoracic echocardiography showing a mobile echodensity within the right atrium (white arrow), attached to a filamentous structure consistent with a Chiari network, raising concern for superimposed vegetation.

**Figure 3:**
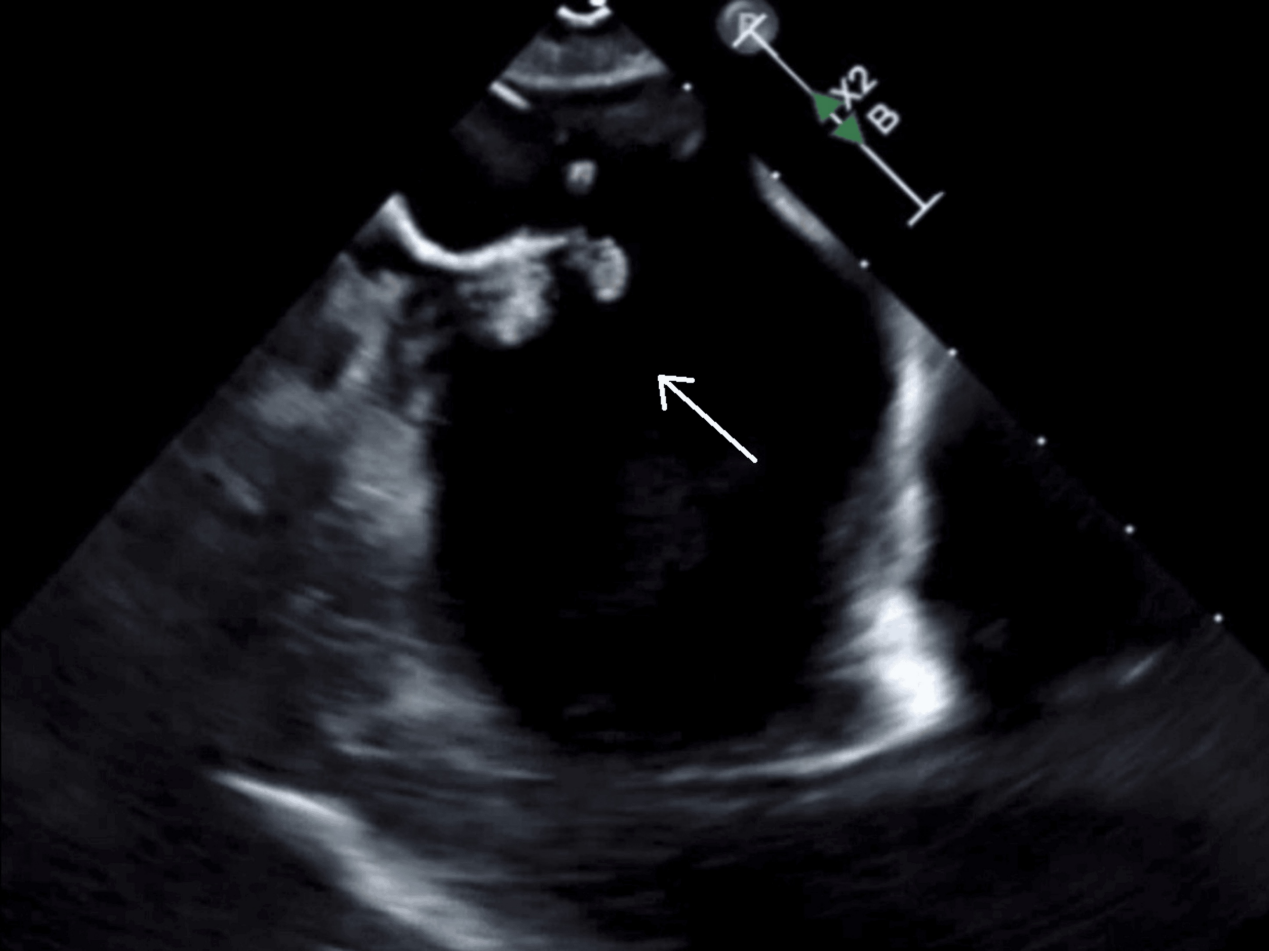
Transthoracic echocardiography (TEE) demonstrating an oscillating mass attached to Chiari network Transesophageal echocardiography demonstrating a prominent Chiari network with an attached oscillating echodense mass (white arrow), consistent with vegetation formation.

No significant valvular abnormalities were identified, aside from trace regurgitation. A right atrial echodensity of unclear significance was noted, prompting further evaluation with TEE. The TEE demonstrated a prominent Chiari network with an oscillating echodensity measuring 0.5 cm × 0.4 cm (Videos 2-4, Figures 2-3), which was a new finding compared with a prior TEE performed in 2020 (Video 5).

**Video 2:** Three-dimensional transesophageal echocardiography (TEE) demonstrating a highly mobile, filamentous Chiari network within the right atrium, with attached oscillating echodensities consistent with vegetations

**Video 3:** Mid-esophageal TEE at approximately 45° showing a mobile Chiari network in the right atrium with superimposed echodense vegetations TEE: transesophageal echocardiography

**Video 4:** X-plane transesophageal echocardiographic imaging demonstrating the Chiari network with attached mobile echodensities

**Video 5:** Prior transesophageal echocardiogram demonstrating a Chiari network in the right atrium without associated vegetations, supporting a pre-existing anatomical variant before the development of infective endocarditis

No patent foramen ovale (PFO), atrial septal aneurysm, or atrial septal redundancy was identified. Bronchoscopy was performed, given the pulmonary findings; all bronchoscopic cultures were negative for any organism. An extensive non-infectious workup, including evaluation for malignancy and autoimmune etiologies, was unremarkable. Repeat blood cultures were also negative.

An infectious disease specialist was consulted for antibiotic management. Given the negative culture data but high clinical suspicion for infective endocarditis involving the Chiari network, the patient was initiated on empiric IV antibiotic therapy with vancomycin and ceftriaxone for a planned six-week course. Cardiothoracic surgery was consulted, and surgical intervention was deferred, given the small size of the vegetation and the absence of hemodynamic compromise.

## Discussion

The Chiari network is the result of incomplete reabsorption of the right valve of the sinus venosus during embryonic development. The low-flow environment within the right atrium caused by the Chiari network's fenestrated, reticular structure predisposes to thrombus formation and bacterial seeding [4]. The Chiari network is present in roughly 2-3% of the general population and does not hold clinical significance. However, due to its highly mobile nature, sometimes differentiating from vegetation, thrombi, or tumors or tricuspid valve disruption can be challenging [5,6]. In the literature, the Chiari network has been associated with PFO, causing right-to-left shunt, leading to paradoxical embolism, supraventricular tachyarrhythmias, atrial septal aneurysm, and recurrent cardiac embolic events [7-10].

Our patient is unique in the sense that there was no infectious source identified, based on clinical symptoms of malaise, dyspnea, transient rash, and presentation of multifocal emboli on chest CT, with supporting echocardiography findings, which led to the diagnosis of Chiari network endocarditis. TTE and TEE are both reliable tools for diagnosing Chiari network; however, TEE is considered the gold standard as it provides superior visualization of right atrial structures [3,11,12]. In our case, TTE identified right atrial echodensity but required confirmation with TEE. Comparison with prior TEE imaging was also crucial to identify the lesion appropriately. The presence of multifocal bilateral pulmonary nodular opacities further supported the diagnosis of right-sided endocarditis [13].

There are currently no definitive guidelines for the management of Chiari network endocarditis based on size or extent of the vegetation [3]. The management of Chiari network generally follows the principles of infective endocarditis in most cases [7,11], where prolonged antibiotic therapy is the cornerstone of the treatment, leading to complete resolution on imaging. Surgical intervention may be required in several specific scenarios: first, if the patient continues to be febrile or develops persistent septic emboli despite the initiation of antibiotics [14]; second, if the patient continues to get recurrent infection, for which Chiari network endocarditis is the source [15]; and third, in culture-negative endocarditis, if the causative organism is fungal or resistant to antibiotics [16].

## Conclusions

Chiari network endocarditis is a rare condition that poses significant diagnostic challenges, as it can mimic other right atrial structures, such as thrombus, vegetations, or tumors. This case highlights the importance of considering Chiari network involvement in patients whose infectious workup is non-revealing. When clinical signs and symptoms suggest right-sided endocarditis, TEE is essential and can yield a definitive diagnosis. Early recognition is critical to guide appropriate treatment and improve clinical outcomes.
